# Menstrual cycle and perceived stress predict performance on the mnemonic similarity task

**DOI:** 10.1371/journal.pone.0322652

**Published:** 2025-05-02

**Authors:** Mateja Perović, Michael L. Mack

**Affiliations:** Department of Psychology, University of Toronto, Toronto, Canada; Memorial University of Newfoundland, CANADA

## Abstract

A growing body of literature demonstrates strong effects of ovarian hormones on the hippocampus and adjacent structures. However, resulting impacts on human cognition remain unclear. Addressing this gap, we examine pattern separation ability, a core hippocampal process, across the menstrual cycle using the mnemonic similarity task as a behavioral index (N = 183). We find a non-linear effect of the menstrual cycle, with pattern separation performance peaking in the high-estradiol, late follicular phase and reaching its lowest point during the mid-luteal phase, which is characterized by moderate estradiol and high progesterone levels. Additionally, we find that perceived stress may facilitate pattern separation performance. These results point to the importance of ovarian hormones for human cognition, reveal novel effects of perceived stress on mnemonic similarity task performance, and provide preliminary evidence of possible effects of menstrual cycle phase on neural pathways involved in pattern separation.

## Introduction

Consideration of sex-related variables in neuroscience is crucial for developing a coherent and complete account of human cognition in health and disease [1,2]. The study of the effects of ovarian hormones on cognition presents a key, but long overlooked, building block of this project [3]. The two primary hormones secreted by the ovaries, estradiol (E2) and progesterone (P4), have been shown to have widespread effects on brain structure in both human [4–6] and animal [5,7,8] studies. However, our understanding of their functional effects on human behaviour remains mixed [3].

**Fig 1:**
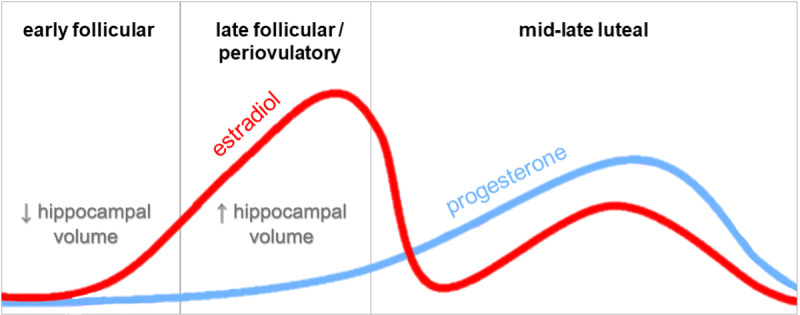
Typical changes in estradiol and progesterone across the menstrual cycle along with associations between menstrual cycle phase and hippocampal structure [14, 16, 17].

One approach to studying the effects of ovarian hormones on human cognition is to leverage their natural increases and decreases across the human menstrual cycle. Specifically, the early follicular phase (EF), beginning at the onset of menses, is characterized by low levels of E2 and P4. E2 levels rise across the late follicular phase, peaking shortly before ovulation. The luteal phase follows. During this phase, levels of E2 decrease significantly, settling to moderate levels as P4 increases during the mid-luteal phase, and decreasing in the late luteal phase (Fig 1; [9]). These predictable changes across the cycle provide a natural manipulation of hormone conditions for researchers interested in a variety of brain networks or structures.

While there are widespread changes in connectivity across the menstrual cycle [10–13], the structure and function of some brain areas seem to be more robustly affected by the menstrual cycle than others. Multiple studies show an association between E2 levels across the cycle and overall hippocampal volume [14–17] (Fig 1), functional connectivity of the hippocampus with other brain regions [15], and hippocampal activity during affective, visuospatial, and verbal processing [16,18,19]. Similarly, hippocampal activity has been found to increase when women in the low-E2 phase of their menstrual cycle are administered E2 resulting in an increase mirroring E2 levels typical of the high-E2 phase of the cycle [20]. Finally, behavioural studies examining hippocampal-dependent tasks report seemingly estradiol-driven variance across the menstrual cycle (e.g., [21–24]).

At least some of the menstrual cycle effects on hippocampal cognition may be explained by E2’s effects on hippocampal pathways involved in pattern separation and completion [25]. Namely, E2 increases synaptic density [26–28] and volume of CA1 [29], which is associated with pattern completion [30]. E2 also potentiates synaptic transmission in CA1, CA3, and dentate gyrus (DG; the latter two of which are associated with pattern separation [30–33]), with the greatest magnitude of potentiation observed in CA3 [34]. These significant structural effects of E2 on neural pathways involved in pattern separation may be reflected in related cognitive performance.

To further probe this hypothesis, we track performance on the mnemonic similarity task (MST), a suggested behavioural index of pattern separation [35], across the menstrual cycle. In this task, participants are shown a series of real-world items and asked to discriminate between “target” items that they have seen earlier in the task, “lure” items which are highly similar to target items, and new, “foil” items. Correct identification of lures is suggestive of the ability to successfully turn new input that is similar to previously stored representations into distinct, non-overlapping representations [30,31,35]. Prior work examining learning exceptions to category rules, a process likely driven by pattern separation processes in the hippocampus [36–38], across the menstrual cycle suggests that successful categorization of exception items is most likely in the high-E2 phase of the cycle [25]. Based on these behavioural findings and animal models of the structural effects of E2 on CA3 and dentate gyrus [34], we expected to see an increase in accuracy for identification of lure items in the late follicular, high-E2, part of the cycle. To this end, we administered the MST to participants across the menstrual cycle, as well as to a male comparison group. We opted to include a male comparison group to account for any potential sex differences in performance, as animal models indicate sex differences in pattern separation performance and strategy [39–41] and human research has suggested that sex differences in pattern separation performance can emerge at specific points in the menstrual cycle [24].

## Methods

### Participants

Participants were recruited through the Prolific online recruiting platform and prescreened for age (22–35), oral contraceptive use, history of mental health diagnosis, history of traumatic brain injury with loss of consciousness, normal or corrected-to-normal vision, and English fluency. A total of 304 participants completed the study. Participants were excluded if they reported any of the following: current use of hormonal contraceptives (n = 19), irregular or “somewhat” regular menstrual cycles (n = 49), cycles outside the typical 21–35 day range (n = 2), not providing any cycle-related information (n = 2), a history of psychiatric disorders (n = 5), if they didn’t identify foil or target items at a rate significantly above chance (62.5%; n = 23) or if over 20% of their reaction times fell outside of the 0.15 – 2s range (n = 15).

The final sample, after exclusions, consisted of 183 participants (age: 26.5 ± 3.9 years, education: 15.98 ± 3.31 years). There were 44 participants in the EF phase, 48 in the LF phase, 48 in the ML phase, and 43 in the male group. Average menstrual cycle length was 28.21 ± 2.5. On average, the EF group was on day 3.73 ± 2.13 of their cycle; the LF group, on day 13.1 ± 2.63; and the ML group, on day 21.6 ± 3.72. The majority (77%) of participants tracked their cycles using an app or a calendar. There were no group differences based on age and education (all p > 0.05). Most participants (n = 108; 59.3%) reported ethnicity of European origin, followed by ethnicity of African origin (n = 39, 21.3%), Latin, Central and South American origin (n = 24, 13.1%), multiple ethnic origins (n = 6, 3.3%), East, Southeast or South Asian origin (n = 5, 2.7%), Caribbean origin (n = 1, 0.5%) and North American Indigenous origin (n = 1, 0.5%).

### Procedure

Participants provided informed consent by selecting a button on the online platform indicating that they consented to taking part in the study. They completed the MST followed by a demographic questionnaire including questions related to their menstrual cycle. Participants received monetary compensation for participation in the study. Recruitment for the study lasted from July 28^th^ until July 30^th^ 2023. All experimental procedures were approved by and conducted in accordance with the University of Toronto Research Ethics Board.

### MST

**Fig 2:**
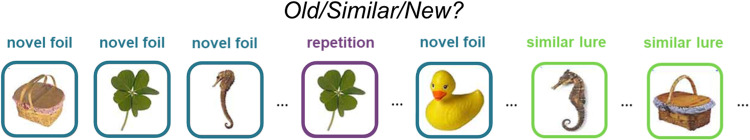
A schematic of the MST task procedure (figure adapted from [ 42]). Participant saw a contiguous sequence of real-world stimuli that were either novel, repeated, or highly similar to previously seen items. For each item, participants indicated whether they thought it was “old”, “new”, or “similar”. The task consisted of 128 trials.

We used the continuous version of the optimized MST [42], an object recognition task sensitive to hippocampal function and typically considered a behavioural measure of pattern separation [35]. Participants viewed a continuous visual presentation of everyday objects that included repeated target items, novel foil items, and lure items which were highly similar to previously seen items (for a schematic of the task structure, see Fig 2). For each item, they responded based on whether they thought the item was “old”, “new” or “similar” to previously seen ones. The ability to correctly identify lure items as similar (rather than mistaking them for previously seen items) indicates successful pattern separation [35,43]. The task consisted of 128 trials.

### Menstrual cycle variables

Our main variable of interest was a continuous measure of participants’ point in the menstrual cycle. We calculated this scaled cycle point variable for each participant by dividing their current day of cycle by their total cycle length. We also estimated each participant’s phase of the menstrual cycle in order to examine possible differences compared to the male group, and to ensure equal representation of different phases of the cycle within our sample. To account for variability in length of menstrual cycles (21–35 days; [44]), menstrual cycle phases were estimated according to each participant’s self-reported cycle length and current day of cycle. The estimated phases, with predicted hormone levels, were as follows: EF – approximately 1–7 days after menses onset, predicted low E2 and progesterone; LF/PO – approximately 8–17 days after menses onset, predicted high E2 and low progesterone; ML – approximately 1–11 days prior to menses onset, predicted moderate E2 and high progesterone.

### PSS

We administered the perceived stress scale (PSS), a commonly used stress-assessment tool [45], due to its previous association with MST performance [46,47], as well as to control for possible interactions between menstrual cycle phase and perceived stress [48]. The scale consisted of ten five-point items (ranging from “Never” to “Very often”) asking about stressful experiences in the month prior (e.g., “how often have you felt nervous and stressed?”). Higher scores indicate higher perceived stress, with scores between 0 and 13 indicating low stress, scores between 14 and 26 indicating moderate stress, and scores between 27 and 40 indicating high stress.

### Statistical analysis

To characterize variation in MST performance across the menstrual cycle, we calculated a standardized variable denoting each participant’s point in their menstrual cycle (cycle point = current day of cycle / cycle length) and modeled its interaction with MST condition (lure, target, foil) in predicting MST performance using generalized additive modeling (GAM). This approach (further outlined in a prior publication, [24]) has several advantages over the more traditional approach of dividing the menstrual cycle into phases and analyzing them as separate groups. It allows for modelling of menstrual cycle effects on cognition in a high-resolution, non-linear fashion that 1) increases statistical power, 2) reduces bias from individual variance in cycle length and 3) captures the full extent of behavioral variance across the menstrual cycle. In a follow-up analysis, we also provide the more traditional, group-based approach by fitting generalized linear models that predict MST performance by group (early follicular, late follicular, mid-late luteal, male) in each of the MST conditions (lure, target, foil). The following dependent measures of MST performance were used in the analyses: overall accuracy, lure discrimination index (p(“Similar” | Lure) – p(“Similar” | Foil) [35]), and rates of “old”, “similar”, and “new” responses.

In order to check if differences in perceived stress emerged across the menstrual cycle, we predicted perceived stress by standardized cycle point using GAM and by menstrual cycle phase using a general linear model. Given that there were no differences between menstrual cycle phases in terms of PSS and following best practices for analyzing mixed-sex samples [2], we ran linear models examining the interaction of PSS and sex on MST performance. Finally, we ran an exploratory GAM examining the interaction between standardized point in cycle and perceived stress.

## Results

### MST across the menstrual cycle

**Fig 3:**
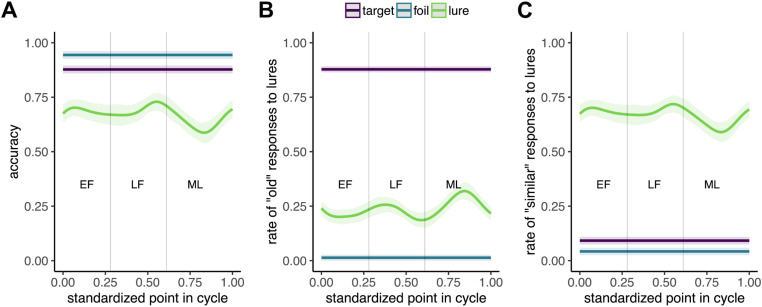
Effects of menstrual cycle on MST performance. **(A)** Overall lure accuracy for the three conditions: foil, target and rule. There is a distinct non-linear effect of cycle point on accuracy for lures, but not for foils or targets, showing a decrease in accuracy in the ML phase of the cycle. **(B)** Rate of responding “old” for each condition. There is a non-linear effect of cycle point on “old” responses for lures, with an increase in ML phase of the cycle. **(C)** Rate of responding “similar” for each condition. There is a non-linear effect of cycle point on “similar” responses for lures, with a slight increase in LF phase and a decrease in ML phase. GAM model estimates are depicted with thick lines; shaded regions depict 95% confidence intervals.

To characterize the difference in MST performance across the menstrual cycle, we modeled the effect of cycle point (cycle point = current day of cycle / cycle length) on overall accuracy for each of the three MST conditions using generalized additive modeling (GAM). Results demonstrated a distinct non-linear effect of cycle point on accuracy for lures (EDF = 4.67, *F*(8) = 188, *p* < .001; Fig 3A), without effects for target or foil items (all EDFs < 1; Note: EDF, or effective degrees of freedom, are a measure of non-linearity in GAM, with an EDF of 1 indicating a linear effect and values higher than 1 indicating non-linearity).

To further explore the mechanism behind changes in lure accuracy across the cycle, we calculated lure discrimination index (LDI) as the difference between responding “similar” to lure items and responding “similar” to foil items: p(“Similar ” | Lure) – p(“Similar ” | Foil) [35] as well as the rate of each type of response (“old”, “similar”, “new”) in each of the three stimulus conditions. We found no effect of menstrual cycle on LDI, but cycle point had significant non-linear effects on both the rate at which participants incorrectly identified lure items as “old” (EDF = 5.12, *F*(8) = 467.8, *p* < 0.001; Fig 3B) and correctly identified them as “similar” (EDF = 4.58, *F*(8) = 190.1, *p* = 0.001; Fig 3C). There were no effects on target or foil items (all EDFs < 1).

A follow-up analysis of MST performance treating the menstrual cycle phases as discrete groups (EF, LF, ML) and comparing them to a male comparison group revealed convergent findings in terms of differences in overall lure accuracy between LF and ML groups (*β* = 0.05, *SE* = 0.03, *t*(179) = 2.04, *p* = .043) as well as differences in “old” responses for lures between EF and ML groups (*β* = 0.04, *SE* = 0.02, *t*(179) = 0.78, *p* = .035). There were no differences between any of the menstrual cycle phase groups and the male group (all p > .05).

### MST and perceived stress

Further, we compared perceived stress scores between our groups, and explored the relationship between perceived stress and classification of lures. There were no significant differences between groups in terms of PSS (EF: 21.2 ± 3.4; LF: 21.6 ± 3.9; ML: 21.6 + 4.9; Male: 20 ± 3.72; S1 Fig). Across all groups, average PSS scores were indicative of moderate perceived stress levels.

**Fig 4:**
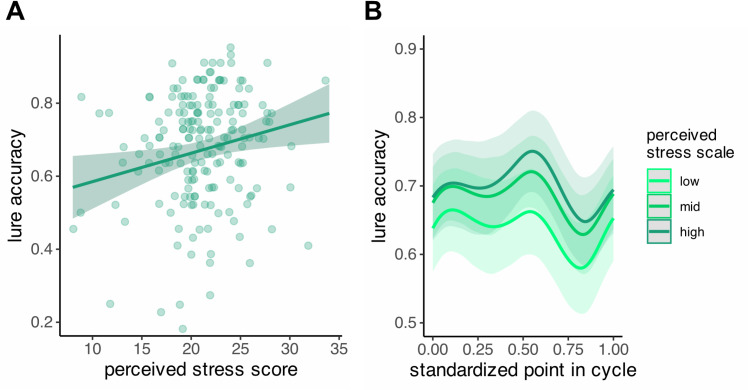
Effects of perceived stress. Positive associations between perceived stress and overall accuracy for lures (A) and lure accuracy across the menstrual cycle by perceived stress score (B). Higher perceived stress is associated with higher lure accuracy. Lure accuracy peaks in the LF phase of the cycle and decreases in ML phase of the cycle. Thick lines represent marginal model estimates; shaded regions represent 95% confidence intervals.

Following best practices for analyzing mixed-sex samples [2], we ran linear models examining the interaction of PSS and sex. We found a positive significant effect of PSS on overall accuracy for lures (*β* = 0.01, *SE* = 0.003, *t*(179) = 2.5, *p* = .01) and lure discrimination index (*β* = 0.01, *SE* = .003, *t*(179) = 2.19, *p* = .03); Fig 4A. The sex by PSS interactions were not significant and there were no main effects of sex (all p > 0.05). To examine whether the PSS results would hold up in a larger sample, we leveraged our full Prolific sample (N = 270) of participants who passed task exclusions and reported no history of psychiatric disorders. Replicating our initial analyses, we found main effects of PSS on overall lure accuracy (β = 0.01, SE = .002, t(266) = 2.23, p = .026) and a marginal effect on LDI (β = 0.01, SE = .002, t(266) = 2, p = .05), such that PSS scores were positively associated with both outcomes.

Additionally, we ran an exploratory generalized additive model examining the interaction between cycle point and perceived stress scale (Fig 4B; S2 Fig), which showed convergent findings with significant main effects of cycle point (EDF = 2.75, *F*(8) = 7.43, *p* = .006) and perceived stress (EDF = 2.12, *F*(3.86) = 3.87, *p* = .018) but no significant interaction (EDF = 1.01, *F*(10) = 2549, *p* = .11).

## Discussion

This is the first study to examine MST performance across the menstrual cycle. We find that the menstrual cycle affects lure accuracy in a distinct, non-linear way, such that lure accuracy peaks mid-cycle, in LF phase, and drops during the ML phase. Rates of “similar” responses to lures show the same pattern, while the rate of “old” responses to lures peaking during the ML phase. This increased tendency to mistake lures for previously seen items in the ML phase explains the overall drop in accuracy for lures during the same phase. The overall pattern for lure accuracy persists when perceived stress, another factor affecting MST performance, is accounted for in the model.

The finding that lure accuracy peaks in the high-estradiol LF phase is consistent with previous literature suggesting that estradiol affects hippocampal structures associated with pattern separation, which are thought to underlie MST performance. Specifically, neuroimaging studies have demonstrated that DG/CA3 respond to similar lures as if they were new items during the MST [31], indicative of their role in pattern separation. The effect has been replicated with use of highly similar lures [30,32], while high-resolution imaging studies have suggested that the effect is localized to DG [33]. Critically for our findings, overall hippocampal volume increases with high E2 across the human menstrual cycle [14–17], and animal studies provide evidence that E2 potentiates synaptic transmission in CA1, CA3, and DG [34]. Therefore, E2 might be boosting lure accuracy through its action on pertinent hippocampal subfields.

On the other hand, lure accuracy drops significantly in the ML phase, when E2 levels are moderate and P4 levels are high. This could be explained by P4’s effects on the medial temporal lobe (MTL) regions involved in pattern separation. Not only does P4 interrupt proliferative effects of E2 on hippocampal dendritic spines in animal models [49] but its levels are negatively associated with perirhinal and entorhinal gray matter volume in humans [11]. The latter two regions may be critical for MST performance as both play a role in upstream resolution of interference between overlapping stimuli, which precedes finer-grained discrimination in the hippocampus [50,51]. Degradation in perforant input from the entorhinal cortex to DG has been linked to deficits in pattern separation [52,53], while the perirhinal cortex has been shown to play a major role in perceptual discrimination of complex objects with a large number of overlapping features [54–57]. Hence, P4 may be modulating the ability of key MTL regions to transmit necessary signals to the hippocampus.

An alternative, or additional, explanation could lie in the fact that increased integration of multiple brain networks is necessary for successful memory encoding [58,59]. Previous work has shown that E2 facilitates and P4 reduces coherence across multiple cortical networks over the course of the menstrual cycle [11]. However, several studies also show that P4 facilitates connectivity between fronto-parietal [60], as well as prefrontal, sensory and hippocampal regions [61] across the cycle, suggesting that the negative effect on lure discrimination that we see in the current study might not be originating from cortical regions and might instead emerge in the MTL. Future studies that incorporate hormonal and/or neuroimaging analyses may adjudicate these potential accounts.

Finally, we administered the perceived stress scale in order to control for possible interactions between menstrual cycle phase and perceived stress [48], which could have been especially important as higher perceived stress has been associated with worse lure discrimination on the MST [46,47]. Consistent with previous longitudinal work on the relationship between menstrual cycle phase and psychological measures [62], we found no effect of cycle point or menstrual cycle phase on perceived stress. However, perceived stress was positively associated with lure accuracy and LDI, with this result replicating in our larger sample.

These findings are in line with research suggesting that moderate stress can improve cognitive function and promote resilience in both human [63–65] and animal models [66,67], including promoting memory consolidation [68,69]. However, previous work on perceived stress and MST with comparable sample sizes but overall lower PSS scores than in our sample [47] initially found no effect of perceived stress on lure discrimination, with differences emerging once an interaction between perceived stress and anhedonic depression was introduced (i.e., the negative association between perceived stress and lure discrimination was only present for participants low in anhedonic depression). This discrepancy in results may suggest a context-dependent effect of PSS on MST performance, and underscores the importance of considering individual differences in psychological factors that may affect cognitive performance. As the sample in that study was balanced in terms of sex and our sample consisted mostly of female participants, it is also possible that these differing results are driven by a sex-specific effect.

Strengths of the current paper include sampling across the entire menstrual cycle and using a continuous cycle point measure. Most published studies on cognition across the menstrual cycle compare only two phases and are thus not able to provide a comprehensive overview of changes across the menstrual cycle (see [70] for a review and critique). Division of the cycle into discrete phases may also reduce predictive power and obscure changes within the phases themselves. Our non-linear GAM approach allowed us to characterize behaviour continuously as it evolved across the cycle. Additional strengths include only using data from participants who reported regular menstrual cycles, and the fact that the majority of participants used an app or calendar to track their cycle.

As the study was conducted online, its main limitation was that E2 and P4 were not directly measured. However, self-reported menstrual cycle information has been shown to align with serum hormone levels [71] and has been successfully used to examine cognitive differences across the menstrual cycle in large online samples [20,23,24]. Additionally, our GAM approach was data-driven and blind to any conceptual hypotheses we had about where differences in performance across the cycle should emerge. The fact that the observed changes tracked so closely with when E2 levels and P4 levels typically peak provides strong evidence for a hormonal effect underlying our findings.

Our work demonstrates novel evidence of menstrual cycle effects on pattern separation performance, likely driven by complex and interacting effects of E2 and P4 on the hippocampus and extra-hippocampal MTL structures. These findings also provide a conceptual replication of previous work implicating E2 in cognitive processes dependent on hippocampal pattern separation pathways [25]. Future research would benefit from examining MST performance in users of oral contraceptives, which suppress endogenous E2 and P4. While there are no studies on oral contraceptive use and pattern separation, studies using other hippocampal-associated tasks suggest that performance under oral contraceptive use is similar to that seen in high-estradiol phases of the menstrual cycle [72]. However, it is important to note that effects of oral contraceptives on cognition vary significantly by type, hormonal composition, and hormone potency [72,73]. Finally, as the menstrual cycle has been shown to predict sub-field-dependent changes in the MTL in high resolution neuroimaging studies [29,74], it may provide a unique avenue for researchers interested in how subtle structural differences in hippocampal subfields affect behavior.

## Supporting information

S1 Fig Null relationships between perceived stress score and (A) standardized point in cycle and (B) menstrual cycle phase. (TIF)

S2 Fig Model predictions from Fig 4B broken down into separate panels. (TIF)
