# Supplementary figures and images for: Menstrual cycle and perceived stress predict performance on the mnemonic similarity task
Source: PLoS One. 2025 May 2;20(5):e0322652. doi: 10.1371/journal.pone.0322652 (PMC12047775; doi:10.1371/journal.pone.0322652)

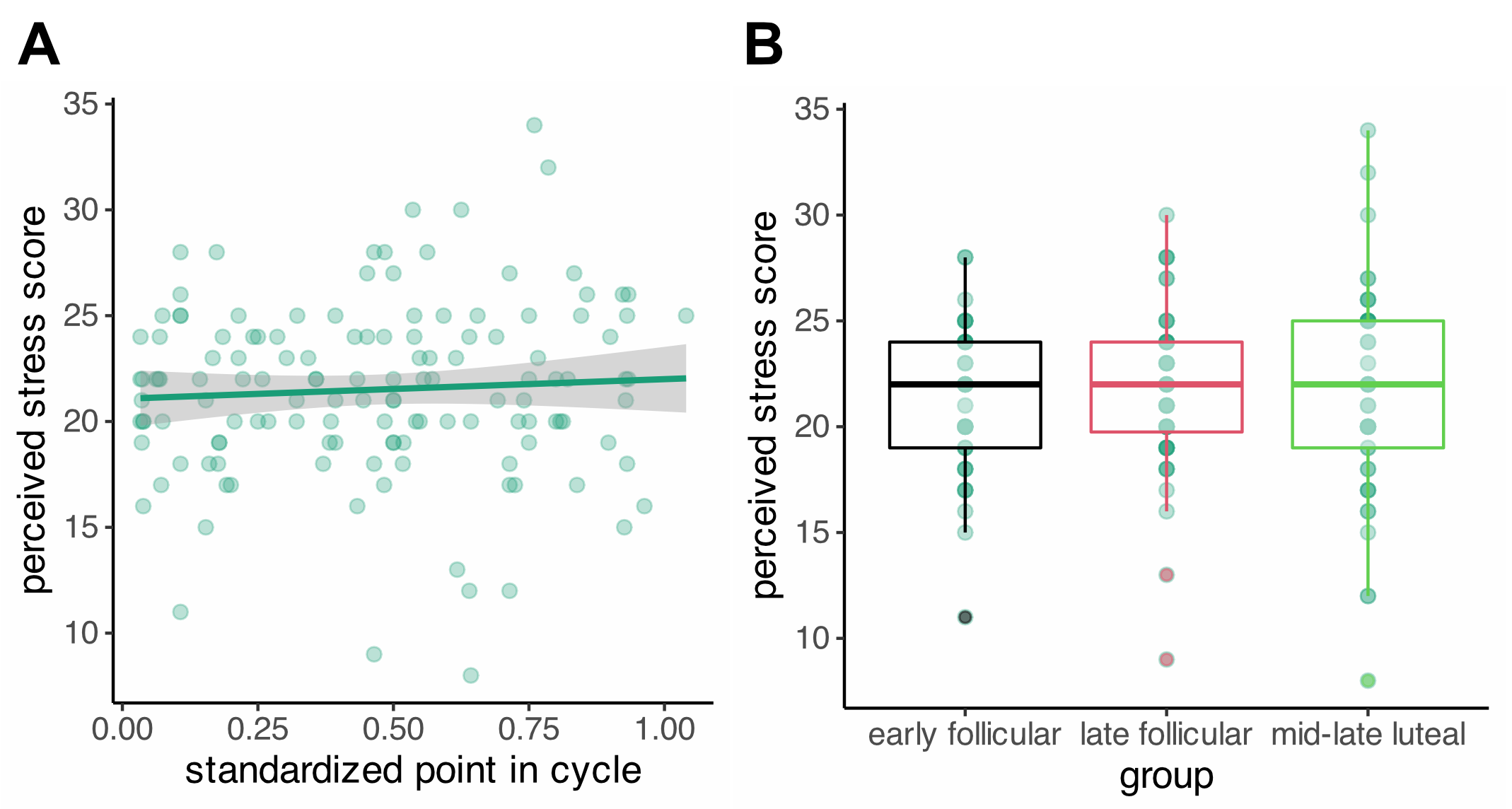

Supplement: S1 Fig — (TIF) [file pone.0322652.s001.tif]

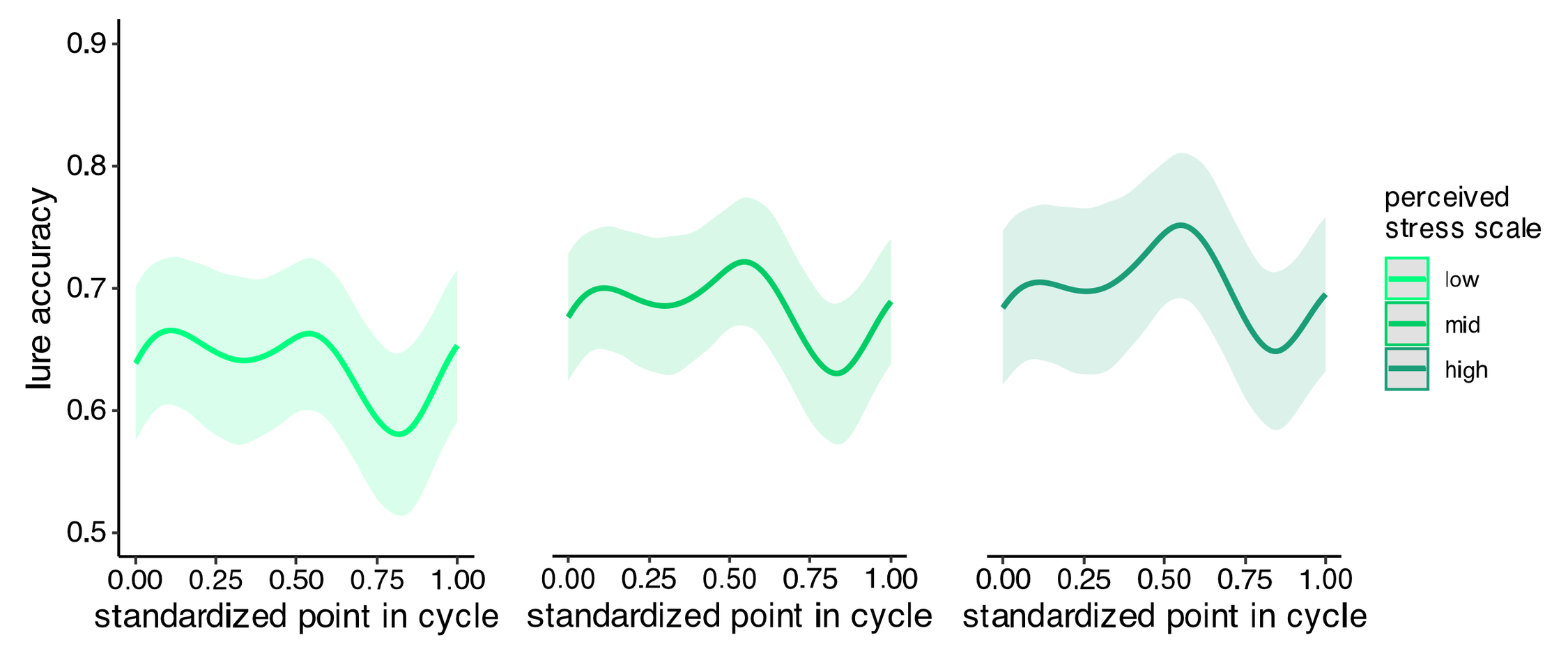

Supplement: S2 Fig — (TIF) [file pone.0322652.s002.tif]
